# Behavioral, Anti-Inflammatory, and Neuroprotective Effects of a Novel FPR2 Agonist in Two Mouse Models of Autism

**DOI:** 10.3390/ph15020161

**Published:** 2022-01-28

**Authors:** Claudia Cristiano, Floriana Volpicelli, Marianna Crispino, Enza Lacivita, Roberto Russo, Marcello Leopoldo, Antonio Calignano, Carla Perrone-Capano

**Affiliations:** 1 Department of Pharmacy, School of Medicine and Surgery, University of Naples Federico II, 80131 Naples, Italy; claudia.cristiano@unina.it (C.C.); roberto.russo@unina.it (R.R.); antonio.calignano@unina.it (A.C.); perrone@unina.it (C.P.-C.); 2 Department of Biology, University of Naples Federico II, 80126 Naples, Italy; crispino@unina.it; 3 Department of Pharmacy-Drug Sciences, University of Bari Aldo Moro, 70125 Bari, Italy; enza.lacivita@uniba.it (E.L.); marcello.leopoldo@uniba.it (M.L.); 4 Institute of Genetics and Biophysics “Adriano Buzzati Traverso”, CNR, 80131 Naples, Italy

**Keywords:** autism spectrum disorders, BTBR, VPA, behavior, neuroinflammation, hippocampus

## Abstract

Autism spectrum disorders (ASD) are a group of heterogeneous neurodevelopmental conditions characterized by social deficits, repetitive stereotyped behaviors, and altered inflammatory responses. Accordingly, children with ASD show decreased plasma levels of lipoxin A4 (LXA4), a mediator involved in the resolution of inflammation, which is the endogenous ligand of the formyl peptide receptor 2 (FPR2). To investigate the role of FPR2 in ASDs, we have used a new ureidopropanamide derivative able to activate the receptor, named MR-39. The effects of MR-39 (10 mg/kg, for 8 days) on hippocampal pro-inflammatory profile, neuronal plasticity, and social behavior were evaluated in two validated animal models of ASD: BTBR mouse strain and mice prenatally exposed to valproic acid (VPA). Primary cultures of hippocampal neurons from BTBR mice were also used to evaluate the effect of MR-39 on neurite elongation. Our results show that MR-39 treatment reduced several inflammatory markers, restored the low expression of LXA4, and modulated FPR2 expression in hippocampal tissues of both ASD animal models. These findings were accompanied by a significant positive effect of MR-39 on social behavioral tests of ASD mice. Finally, MR-39 stimulates neurite elongation in isolated hippocampal neurons of BTBR mice. In conclusion, these data indicate FPR2 as a potential target for an innovative therapeutical approach for the cure of ASD.

## 1. Introduction

Autism spectrum disorders (ASD) are a heterogeneous set of childhood neurodevelopmental disorders characterized by persistent deficits in social communication and interactions, as well as restricted and stereotyped behavior and interests [1,2,3,4]. Although ASD have complex and still controversial pathogenesis, the consensus is that these disorders are linked to both genetic and environmental risk factors [5]. Recent studies repeatedly reported that one of the most common risk environmental factors associated with ASD is ongoing neuroinflammation in various brain regions [6,7]. ASD patients display altered inflammatory states and immune abnormalities throughout their life [8,9], and post-mortem studies on the brains of autistic subjects have shown increased levels of pro-inflammatory cytokines and microglia activation [10,11].

The persistent neuroinflammatory state is often associated with cognitive and behavioral changes that mimic autism [12]. On the other hand, ASD symptoms seem to be linked to an inflammatory state and altered immune functions. It is noteworthy that, due to the bidirectional communication between the immune system and the brain [13], cytokine levels are able to modulate neuronal function and behavioral processes [14]. Moreover, multiple lines of evidence from genetic linkage studies on animal models have highlighted aberrant brain plasticity in the pathophysiology of ASD, including altered neurogenesis, neurite outgrowth, synaptogenesis, and synaptic plasticity [15,16,17]. In this scenario, a key role is played by the hippocampus, a brain region where active neurogenesis and long-term changes of synaptic strength take place [18,19]. Indeed, it has been demonstrated that deficits in this brain area are related to ASD [20]. Interestingly, it was also reported that improving hippocampal neurogenesis and reducing neuroinflammation and oxidative stress can contribute to restoring the normal social behavioral phenotypes of ASD animal models [16,21,22].

Various animal models have been used to mimic ASD symptoms and to study the involvement of inflammation in the pathology. Among them, the inbred BTBR T + Itpr3tf/J (BTBR) mouse strain is one of the most widely utilized, since it displays a range of behavioral deficits characterizing ASD: low levels of sociability, altered communication, and repetitive/compulsive behaviors [23]. Furthermore, BTBR mice have dysfunction of both the innate and adaptive responses of the immune system, and an inflammatory immune profile resembling ASD [24].

About the pharmacological/environmental models of ASD, the prenatally-exposed valproic acid (VPA) model is the most widely used since it displays some behavioral, cognitive, and neuroanatomical alterations characterizing ASD [25,26,27]. In addition, it shows long-lasting alterations in both peripheral and brain inflammatory responses linked to reduced sociability [28], similar to the response of children prenatally exposed to VPA [29]. Moreover, in the adult brains of both VPA and BTBR mice, a reduced hippocampal neurogenesis was observed [30,31].

A growing number of studies have demonstrated the potential beneficial effects of lipids in inflammatory processes. Lipoxins (LXs) are a group of endogenous lipid mediators, which together with resolvins, protectins, and maresins, contribute to controlling inflammation [32,33,34,35]. LXs exert neuroprotective and analgesic effects [36,37,38] in different animal models of peripheral and central disorders, including cardiovascular diseases, traumatic brain injury, and neuropathic pain [39,40,41,42]. However, their anti-inflammatory effects in other nervous system diseases, such as neurodegenerative and neurodevelopmental disorders, are poorly investigated.

Among LXs, Lipoxin A4 (LXA4) has potent anti-inflammatory properties, such as the inhibition of the production of pro-inflammatory cytokines, the suppression of the activities of metalloproteinases, and the stimulation of the clearance ability of macrophages, through the activation of formyl peptide receptor 2 (FPR2). In detail, the activation of FPR2 by LXA4 triggers different cell-specific signaling pathways, as for instance the inhibition of calcium-calmodulin dependent protein kinase and p38 mitogen-activated protein kinase phosphorylation [35,43,44], the inhibition of nuclear factor-κB (NF-κB) [45] and activator protein 1 (AP-1) [46], or the induction of the suppressor of cytokine signalling-2 [47].

FPR2, also known as FPRL1 or ALX/FPR2, belongs to the family of formyl peptide receptors, along with FPR1 and FPR3 [48], and it is broadly expressed in different types of cells, including some immune cells, astrocytes, and neurons in several brain areas [49]. It is a nonconventional receptor not only because it binds different ligands but also because its activation results in various and opposite effects (pro- or anti-inflammatory) depending on the ligand [50,51,52]. As mentioned before, LXA4 and its analogue aspirin-triggered lipoxin are the most important endogenous agonists, with anti-inflammatory and pro-resolving profiles [42]. Interestingly, a link has been demonstrated between LXs and autism. In particular, Yan and co-workers found significantly lower plasma levels of LXA4 in autistic Chinese children compared with normal children. The authors observed a significant negative correlation between circulating LXA4 levels and the severity of autism, suggesting an increased susceptibility to recurring inflammation in these subjects [53].

Therefore, we hypothesized that these lipid mediators may represent a novel strategy to control the excessive neuroinflammation characterizing ASD and may also have beneficial effects on altered behavior associated with the pathology. Unfortunately, LXA4 and its analogues have unfavorable pharmacokinetic properties, which represent a limitation for in vivo studies and clinical trials. In particular, LXA4 is inactivated in microglial cells by dehydrogenation to 15-oxo-lipoxin A4 [42,54].

To circumvent this problem, we have recently synthesized different FPR2 agonists [55,56] and selected one of them, named MR-39, for our current study. MR-39 shows favorable pharmacokinetic characteristics and displays strong anti-inflammatory activities in lipopolysaccharide (LPS)-stimulated rat primary microglial cells, reducing interleukin-1β (Il-1β) and Tumor necrosis factor-α (Tnf-α) levels; in addition, it has a good permeation rate in hCMEC/D3 cells, an in vitro model of the blood–brain barrier [57], and alleviates the inflammatory process associated with Alzheimer diseases [58].

In the present work, we investigated for the first time the potential effects of the FPR2 agonist MR-39 in two ASD mouse models: a polygenic model represented by the BTBR inbred mouse strain, and a pharmacological VPA-induced mouse model. We tested the impact of MR-39 on the hippocampal neuro-inflammatory profile, as well as on stereotyped and social behaviors in both ASD mouse models. Finally, to address the modulation of neuroplasticity in response to FPR2 activation, we evaluated the MR-39 effect on neurite elongation in neuronal primary cultures obtained from the hippocampus of the BTBR mouse strain.

## 2. Results

### 2.1. MR-39 Modulates the Expression of Lipoxin A4 and Its Receptor FPR2 in ASD Mouse Models

**Figure 1:**
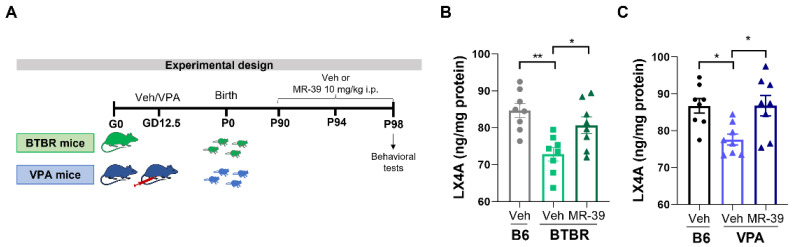
Effect of MR-39 on hippocampal LXA4 level in BTBR and VPA mice. (**A**) Experimental design. (**B**) Hippocampal LXA4 concentration (ng/mg protein) in B6 or BTBR mice intraperitoneally injected for 8 days with 10 mg/kg MR-39 or vehicle for 8 days (*n* = 8 per group). (**C**) Hippocampal LXA4 concentration (ng/mg protein) in B6 or VPA mice intraperitoneally injected for 8 days with 10 mg/kg MR-39 or vehicle for 8 days (*n* = 8 per group). Results are shown as mean ± s.e.m. Differences were evaluated by ANOVA followed by Tukey’s post hoc multiple comparisons, * *p* < 0.05, ** *p* < 0.01.

LXA4, besides showing a strong anti-inflammatory activity, is also potentially relevant for the pathophysiology of ASD [53]. Thus, we measured by ELISA the relative level of LXA4 in the hippocampus of two mouse models of ASD, and we observed a significant reduction of LXA4 in BTBR compared to B6 mice (Figure 1B,C). Interestingly, when BTBR mice were subjected to i.p. injection with 10 mg/kg MR-39 for 8 days, the decrease in the LXA4 level was reverted (Figure 1B, F(2, 21) = 8.987, *p* = 0.0015, one-way ANOVA). Additionally, in VPA mice, the endogenous hippocampal level of LXA4 was decreased compared to B6 mice, and the treatment of these animals with MR-39 significantly increased the LXA4 hippocampal level (Figure 1C, F(2, 21) = 6.082, *p* = 0.0082, one-way ANOVA). This chronic treatment was chosen based on the results of experiments evaluating the behavioral response of animal models to different doses of MR-39 administered for different times (see Supplementary material).

**Figure 2:**
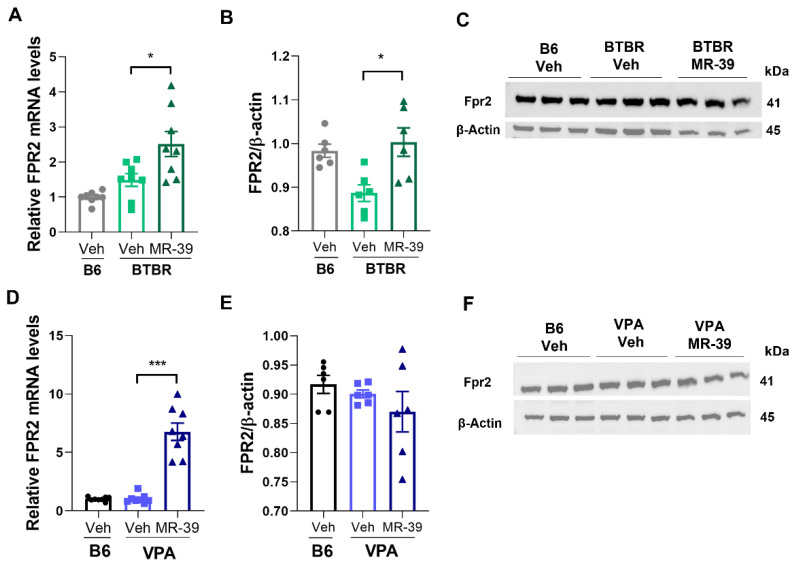
MR-39 modulation of hippocampal FPR2 mRNA and protein expression in BTBR and VPA mice. Expression levels of FPR2 mRNAs, determined by real-time RT-PCR, in hippocampus of B6 and BTBR mice (**A**) or VPA mice (**D**) intraperitoneally injected for 8 days with 10 mg/kg MR-39 or vehicle (*n* = 6 per group). The FRP2 mRNA levels were normalized with those of the housekeeping gene (GAPDH) (2^−ΔΔCT^ method); the bar represents the folds of FPR2 level compared with the B6-vehicle group. Quantitation of FPR2 protein, by western blot analysis, in hippocampus of BTBR (**B**) or VPA (**E**) mice intraperitoneally injected for 8 days with 10 mg/kg MR-39 or vehicle. The bars represent the densitometric values of FPR2 signals normalized with those of β-actin (*n* = 6 per group). Results are shown as mean ± s.e.m. Differences were evaluated by ANOVA followed by Tukey’s post hoc multiple comparisons, * *p* < 0.05, *** *p* < 0.001. (**C**,**F**) Representative images of western blot analysis (*n* = 3) in hippocampus of BTBR (**C**) or VPA (**F**) mice intraperitoneally injected for 8 days with 10 mg/kg MR-39 or vehicle. The molecular weight of the analyzed proteins expressed in kDa is indicated on the right.

To further investigate the role of LXA4 in ASD animal models, we also examined the expression of the LXA4 receptor FPR2 in the hippocampus. The results showed that both BTBR and VPA mice have the same endogenous level of FPR2 mRNA and protein compared to their respective B6 mice (Figure 2). When BTBR and VPA mice were i.p. injected with 10 mg/kg MR-39 for 8 days, the FPR2 mRNA expression level significantly increased in both mice models (Figure 2A, F(2, 21) = 11.07, *p* = 0.05, one-way ANOVA and Figure 2D, F(2, 21) = 57.33, *p* = 0.0001, one-way ANOVA), while the FPR2 protein expression significantly increased in BTBR mice (Figure 2B F(2, 15) = 7.091, *p* = 0.0068, one-way ANOVA and Figure 2C), but not in VPA mice (Figure 2E F(2, 15) = 1.147, *p* = 0.3439, one-way ANOVA and Figure 2F). The discrepancy between mRNA and protein levels in the VPA model could be due to a delay between transcriptional induction and protein level increase, as previously reported [59].

### 2.2. The FPR2 Agonist MR-39 Modulates Hippocampal Cytokines in ASD Mouse Models

**Figure 3:**
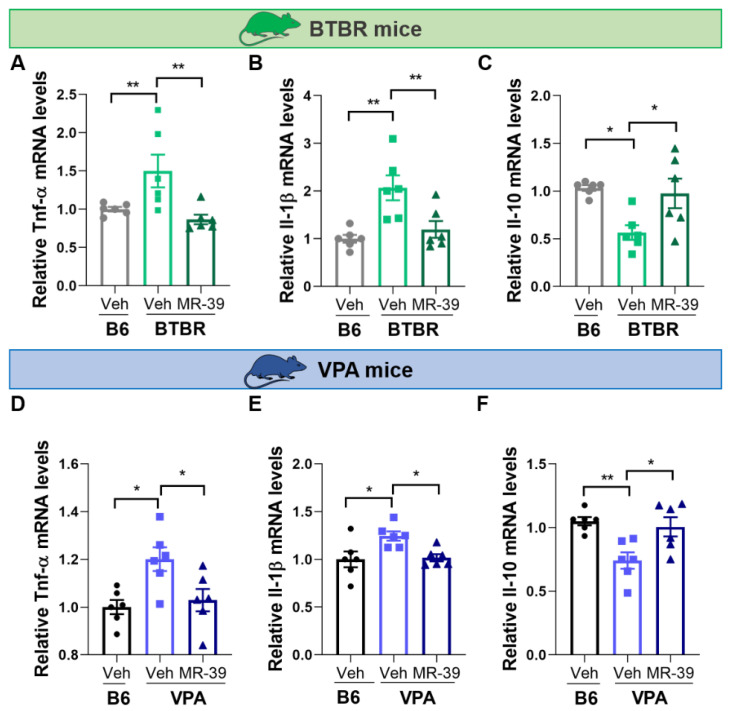
MR-39 modulation of hippocampal pro- and anti-inflammatory cytokine mRNAs. Expression levels of mRNAs for pro-inflammatory Tnf-α (**A**) and Il-1β (**B**), and anti-inflammatory Il-10 (**C**) in hippocampus of B6 and BTBR mice injected intraperitoneally for 8 days with 10 mg/kg MR-39 or vehicle (*n* = 6 per group). Expression levels of mRNAs for pro-inflammatory Tnf-α (**D**) and Il-1β (**E**), and anti-inflammatory Il-10 (**F**) in the hippocampus of B6 and VPA mice injected intraperitoneally for 8 days with 10 mg/kg MR-39 or vehicle (*n* = 6 per group). The mRNA levels were normalized with those of the housekeeping gene GAPDH (2^−ΔΔCT^ method); the bar represents the fold change of each mRNA level compared with the B6-vehicle group. Results are shown as mean ± s.e.m. Differences were evaluated by ANOVA followed by Tukey’s post hoc multiple comparisons, * *p* < 0.05, ** *p* < 0.01.

To assess the anti-inflammatory and pro-resolving effects of the FPR2 agonist MR-39 in the hippocampus of BTBR mice, the animals were i.p. injected with 10 mg/kg MR-39 for 8 days. Then, the hippocampal mRNA levels of the pro-inflammatory factors Tnf-α, and Il-1β, and the anti-inflammatory factor Il-10 were analyzed by real-time PCR. As shown in Figure 3, BTBR mice—compared to B6 mice—showed a significant increase of Tnf-α and Il-1β mRNA levels in the hippocampus, while Il-10 expression was significantly decreased. Interestingly, when the BTBR mice were i.p. injected with 10 mg/kg MR-39 for 8 days, a significant reduction of Tnf-α (Figure 3A, F(2, 15) = 6.651, *p* = 0.0086) and Il-1β (Figure 3B, F(2, 15) = 9.180, *p* = 0.0025) levels, and an increased level of Il-10 (Figure 3C, F(2, 15) = 6.266, *p* = 0.0105) was observed in the hippocampus. Thus, MR-39 was able to restore the expression levels of Tnf-α, Il-1β, and Il-10 to those measured in vehicle-treated B6 mice.

Similarly, in the hippocampus of VPA-exposed mice, the mRNA levels of pro-inflammatory cytokines Tnf-α, and Il-1β were significantly upregulated, while the mRNA level of anti-inflammatory cytokine Il-10 was significantly downregulated compared with B6 mice. Moreover, in this mouse model, the i.p treatment with 10 mg/kg MR-39 for 8 days was able to significantly reduce the Tnf-α (Figure 3D, F(2, 15) = 6.368, *p* = 0.0100) and Il-1β (Figure 3E, F(2, 15) = 5.389, *p* = 0.0172) mRNA levels, and to increase the mRNA level of Il-10 (Figure 3F, F(2, 15) = 7.650, *p* = 0.0051) in the hippocampus. These results demonstrated that MR-39 exerts anti-inflammatory and pro-resolving effects in the hippocampus of both ASD mouse models through stimulation of FPR2.

### 2.3. The FPR2 Agonist MR-39 Increased Sociability of BTBR Mice

The effect of MR-39 on ASD-like behavior was investigated by analyzing both the repetitive/perseverative phenotype by marble burying and self-grooming tests (Supplementary material), and the animal sociability by the 3-chambered social test and the reciprocal social interaction test.

**Figure 4:**
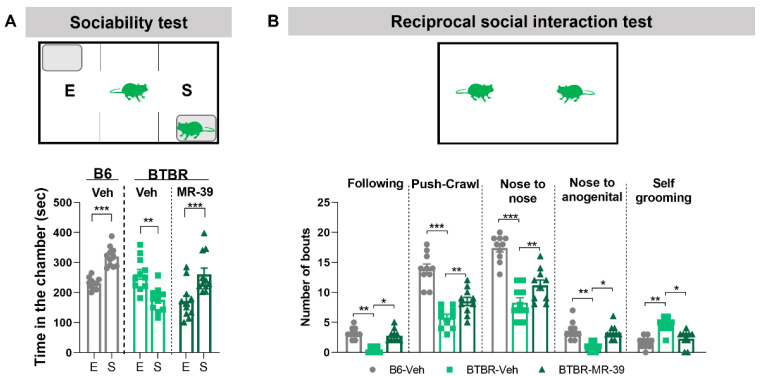
MR-39 reverses the altered social behavior of BTBR mice. (**A**) Time (in seconds) spent in each chamber, during the 10 min three-chambered social test, and (**B**) number of bouts shown, in the 20 min reciprocal social interaction test, by B6 and BTBR mice intraperitoneally injected for 8 days with 10 mg/kg MR-39 or vehicle. E: chamber with novel object; S: chamber with novel mouse. Results are shown as mean ± s.e.m. (*n* = 10–12). (**A**) Differences were evaluated by Student’s *t* test, ** *p* < 0.01, *** *p* < 0.001. (**B**) Differences were evaluated by ANOVA followed by Tukey’s post hoc multiple comparisons, * *p* < 0.05, ** *p* < 0.01, *** *p* < 0.001.

When B6 and the BTBR mice were exposed to the three-chambered social test, the B6 mice, which are extensively social, spent more time in the chamber with the novel mouse than in the chamber with the novel object. On the contrary, vehicle-treated BTBR mice spent significantly less time in the chamber with the novel mouse than in the chamber with the novel object (Figure 4A), and this altered behavior was completely reverted by i.p. administration of MR-39 (10 mg/kg, for 8 days). Indeed, the MR-39 BTBR mice spent significantly more time in the chamber with the novel mouse than in the one with the novel object (Figure 4A, F(5, 60) = 15.25, *p* = 0.0064, one-way ANOVA). Consistently, in the reciprocal social interaction test, vehicle-treated BTBR mice showed a lower number of following, push-crawl, nose to nose, and nose to anogenital sniffing and increased self-grooming compared with B6 mice. When BTBR mice were i.p. injected with MR-39 (10 mg/kg, for 8 days) the number of following, push-crawl, nose to nose, and nose to anogenital sniffing increased significantly, while self-grooming decreased compared to BTBR-vehicle group, suggesting more interest of the treated animals to familiarize and to the social contest (Figure 4B, F(14, 135) = 86.32, *p* < 0.0001, one-way ANOVA). Interestingly, this behavioral data parallels our results on the inflammatory profile of BTBR animals, confirming the link between inflammation and impaired sociability [60].

It is noteworthy that the MR-39 treatment was not effective in rescuing the self-grooming behavior of BTBR animals when the mouse was alone in the cage (see Figure S1b), while it significantly decreased this stereotyped behavior when two mice were together in the cage mouse, suggesting the MR-39 rescuing effect depends on the social interaction.

### 2.4. The FPR2 Agonist MR-39 Increased Sociability of VPA Mice

**Figure 5:**
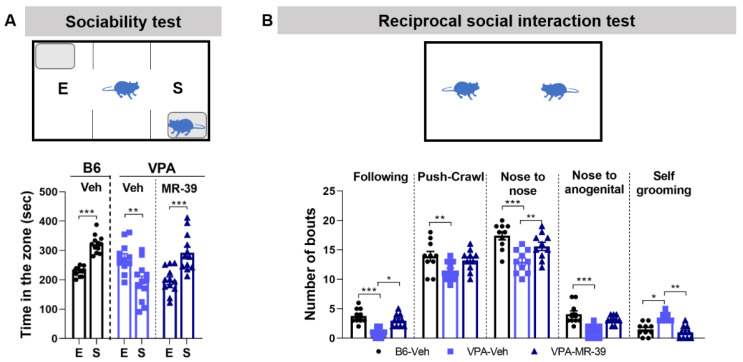
MR-39 reverses the altered social behavior of VPA mice. (**A**) Time (in seconds) spent in each chamber, during the 10 min three-chambered social test, and (**B**) number of bouts shown, in the 20 min reciprocal social interaction test, by B6 and VPA mice intraperitoneally injected for 8 days with 10 mg/kg MR-39 or vehicle. E: chamber with novel object; S: chamber with novel mouse. Results are shown as mean ± s.e.m. (*n* = 10–12). Differences were evaluated by Student’s *t* test, ** *p* < 0.01, *** *p* < 0.001 (**A**), and by ANOVA followed by Tukey’s post hoc multiple comparisons, * *p* < 0.05, ** *p* < 0.01, *** *p* < 0.001 (**B**).

Behavioral tests were also performed in the VPA mouse model. As for the BTBR mice, VPA mice treated i.p. with 10 mg/kg MR-39 for 8 days showed a significant increase of sociability in the three-chambered social test compared to the VPA-vehicle group (Figure 5A, F(5, 66) = 14.51, *p* < 0.0001, one-way ANOVA). During the reciprocal social interaction test, similar to BTBR animals, vehicle VPA mice displayed a lower number of following, push-crawl, nose to nose, and nose to anogenital sniffing, and a higher number of self-grooming compared to B6 mice. The i.p. treatment with 10 mg/kg MR-39 for 8 days increased the number of following and nose to nose sniffing and decreased the time spent in licking itself compared to the VPA-vehicle group (Figure 5B, F(14, 135) = 151.5, *p* < 0.0001, one-way ANOVA). As for the BTBR mice, the results of self-grooming behavior on VPA mice also confirm in this strain that social interaction is critical for the MR-39 rescuing effect on stereotyped behavior.

### 2.5. The FPR2 Agonist MR-39 Selectively Stimulates Neurite Outgrowth in Hippocampal Neurons of BTBR Mice

**Figure 6:**
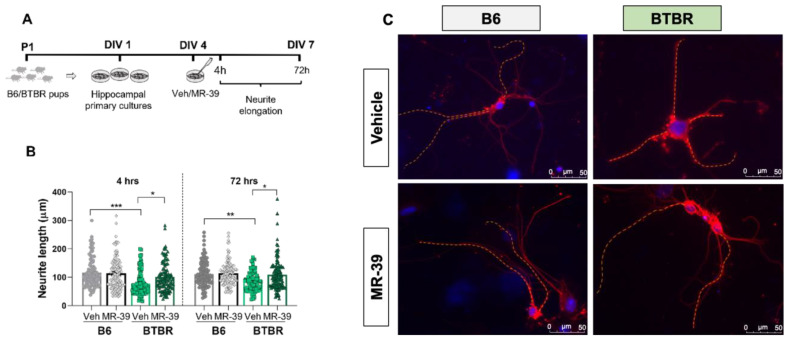
MR-39 selectively stimulates neurite outgrowth in hippocampal neurons of BTBR mice. (**A**) Experimental design. (**B**) Neurite length (µm) of hippocampal neurons from brain of B6 and BTBR mouse pups (P1). Cultures on the 4th day in vitro (DIV) were treated with vehicle (Veh) or FPR2 agonist (MR-39, 10 µM) for 4 h and 72 h. Data are presented as the mean ± s.e.m. from randomly selected fields for each cell culture condition. For each experimental point, cultures were prepared at least in independent triplicate wells, and a total of 150 neurites was analyzed for each cell culture condition. Differences were evaluated by ANOVA followed by Tukey’s post hoc multiple comparisons, * *p* < 0.05, ** *p* < 0.01, *** *p* < 0.001. (**C**) Representative images of hippocampal primary cultures from B6 and BTBR mice treated with vehicle or MR-39 for 72 h. The cells were stained with the neuronal marker β-tubulin III (red) and the nuclear marker DAPI (blue). Scale bar = 50 µm. A dashed yellow line was manually drawn to indicate the neurite length from the soma to the end of the primary neurite.

Neurite elongation, a parameter widely used to evaluate the plastic responses of primary cultured neurons [61,62,63], was examined on dissociated hippocampal cultures obtained from BTBR and B6 pups (P1) and grown for 4 days in a medium without serum to inhibit glial proliferation. Interestingly, we observed that the neurite length of P1 hippocampal neurons in culture was significantly shorter in control BTBR mice than in B6 mice both at 4DIV and at 7DIV (Figure 6B,C).

To evaluate the effect of FPR2 stimulation, hippocampal neurons from B6 and BTBR mice were treated for 4 h and 72 h with MR-39 (10 μM) and the neurite outgrowth was estimated. Remarkably, agonist stimulation of the receptor significantly increased neurite elongation of BTBR hippocampal neurons whose length was restored to B6 levels (Figure 6B, F(7, 828) = 8.694, *p* < 0.0001). It is noteworthy that receptor stimulation did not affect neurite outgrowth of hippocampal neurons from B6 mice (Figure 6B,C).

## 3. Discussion

Although growing evidence supports a close link between dysregulated inflammatory pathways and ASD [64,65], the current literature on the efficacy of anti-inflammatory interventions in ASD is still limited. In this study, we explored the role of a novel ligand of the FPR2, a receptor involved in the resolution of inflammation, on two well-validated models of ASD: the BTBR inbred mouse strain (polygenic model) and mice prenatally exposed to VPA (pharmacological model). These two mouse models, which display the main behavioral, cognitive, and neuroanatomical alterations characteristic of ASD, were chosen because both show long-lasting peripheral and brain inflammatory responses [24,27] and are complementary for their etiology [a genetic model and a pharmacological model] [29]. Indeed, increasing evidence shows that both genetic and pharmacological/environmental factors are deeply involved in the etiopathogenesis of ASD [5,66]. Various studies proposed the modulation of inflammatory pathways as a promising strategy to treat CNS disorders, including ASD [67,68]. FPR2 is involved in different aspects of the inflammatory response, and it has been proposed as a key player in the resolution of inflammation. Nevertheless, endogenous ligands, such as LXA4, do not have favorable pharmacokinetic properties for in vivo studies and clinical trials, making it necessary to identify new molecules [42,57]. Recently, we have identified a series of ureidopropanamide FPR2 agonists with favorable pharmacokinetic characteristics and, among them, we selected for this study the compound MR-39 for its good metabolic stability, passive diffusion, and permeation rates in an in vitro model of the blood–brain barrier. In addition, the MR-39 shows anti-inflammatory and pro-resolving properties in primary microglial cells [57] and in a mouse model of Alzheimer’s disease [58].

To the best of our knowledge, the involvement of FPR2 activation has never been assessed before in ASD animal models. Thus, in the present work, we investigated for the first time the effects of MR-39, an FPR2 agonist, in two different ASD mouse models. In particular, we tested various doses of MR-39, intraperitoneally injected in mice at different time points, and based on behavioral data, we chose a chronic treatment using 10 mg/kg of MR-39, daily injected for 8 consecutive days. In BTBR mice, we observed a significant reduction of LXA4 level, in line with data reported in autistic patients [53]. Interestingly, the treatment of these animals with MR-39 was able to rescue this deficit. In VPA mice, the level of LXA4 was only slightly decreased compared with B6 mice, but also in this case the treatment with MR-39 was able to significantly increase the LXA4 level. Future investigation will be dedicated to analyzing in detail the molecular mechanisms underlying the MR-39-dependent rescue of LXA4 levels in ASD animal models.

Considering that FPR2 is expressed in the CNS and in particular in hippocampal neurons [69], we analyzed the hippocampal mRNA and protein level of this receptor in both mouse models of ASD. Although we did not observe any change in FPR2 expression in animal models compared to their respective control strains, treatment with MR-39 was able to increase the expression of FPR2 in both mice models.

Interestingly, we observed that the administration of MR-39 was able to reduce the expression of Tnf-α and pro-inflammatory Il-1β, and to increase the anti-inflammatory Il-10 in the hippocampus of both BTBR and VPA mice. This profile of anti-inflammatory and pro-resolving responses is well known to be linked to the activation of FPR2 [70]. Thus, these data support the role of MR-39 as a ligand of FPR2 and mediator of its activation.

To identify the possible effect of MR-39 for the treatment of ASD core symptoms, we tested ASD mouse models for stereotyped and social behavior, which are strongly altered in ASD. To analyze the repetitive behaviors, we assessed, in both BTBR and VPA mice, marble burying and self-grooming with the mouse alone in the cage during the task. We did not observe any effect of MR-39 treatment (see Supplementary material). Social behavior was analyzed using both the three-chamber social test and the reciprocal social interaction test. In the first test, MR-39 significantly increased the time that both BTBR and VPA mice spent in the chamber with the novel mouse instead of the chamber with the novel object. In the reciprocal social interaction test, all parameters investigated were significantly modulated in BTBR mice treated with MR-39, while in VPA mice the FPR2 agonist was able to modulate three out of five parameters. One of the parameters examined in the reciprocal social test is the number of self-groomings. Interestingly, in both ASD models, MR-39 was able to reduce self-grooming in this test, unlike what was observed when the mouse was alone in the cage. This diminished self-grooming activity indicates a higher interest of the mouse in socializing, and less focus on itself. These data confirm the importance of social interaction for the MR-39 effect. Altogether, the behavioral results indicate that chronic treatment with MR-39 significantly ameliorates social behavior in both mouse strains, but does not influence repetitive behavior, and suggests that social interaction plays a crucial role in improving the effects of MR-39 on stereotyped behavior of ASD animals.

It is important to consider that social impairments, common to many psychiatric illnesses, are also often associated with an inflammatory state [22,60,67]. Hence, the improvement in social behavior by MR-39 might be linked to the strong anti-inflammatory effect of this agonist in the CNS. On the contrary, repetitive behavior might not be influenced by the body inflammatory level. Accordingly, data on different animal models of ASD indicate that some natural and pharmacological agents are able to reverse both repetitive and social behaviors [71,72] while other agents can reverse only stereotyped [73] or social behaviors [74,75,76,77,78,79]. Alterations of anatomical/functional connectivity are well documented in ASD patients and are likely to contribute to the core phenotypes of impaired social communication and repetitive behaviors. Regional under- and hyper-connectivity reported in children with ASD by functional MRI studies is consistent with abnormalities in neuronal morphology detected in both ASD post-mortem human tissues and animal models [80,81]. In addition, ASD is often associated with the mutation of genes involved in synaptic plasticity [82]. To investigate the plastic remodeling of neurons and their possible alterations related to ASD, we evaluated neurite elongation in primary cultures of hippocampal neurons from BTBR mouse pups and their control B6 mouse strain. Interestingly, we observed that the neurite length of BTBR neurons was strongly reduced compared to B6 mice. This is the first demonstration that BTBR neurons display impaired neuritogenesis that might be associated with brain connectivity deficits in mice models of ASD. Moreover, our results demonstrate that the neurite elongation deficits in BTBR neuronal cultures were significantly improved by the FPR2 agonist MR-39, which was able to bring back the neurite length almost at the level of B6 neurites. These data are in agreement with previous results indicating that resolvin D1, a pro-resolving and anti-inflammatory mediator, ligand of FPR2, stimulates neurite outgrowth in primary cultured dorsal root ganglion cells [83].

## 4. Materials and Methods

### 4.1. Animals

Mice were housed in the animal care facility at the Department of Pharmacy of the University of Naples Federico II, Italy, in a room with controlled temperature (22 ± 1 °C), humidity (60 ± 10%), and light (12 h per day). Food and water were available ad libitum. All behavioral tests were performed between 09:00 and 14:00 h, and the animals were used only once. After completion of the experiments, the animals were sacrificed.

#### 4.1.1. BTBR Mouse Strain

For our studies, BTBR T + tf/J (BTBR) mice and their control C57Bl/6J mice (B6) were purchased from the Jackson Laboratory (Bar Harbor, ME, USA). We used 3-month-old male mice (*n* = 10–12) housed in groups in the same room under controlled temperature and humidity, on a 12 h:12 h light:dark cycle, with ad libitum access to water and a standard laboratory chow diet. Male pups of the B6 and BTBR mice were used for neuronal primary cultures.

#### 4.1.2. VPA Mouse Model

The prenatally exposed VPA animal model of autism (VPA mice) was induced as previously described [84]. Briefly, adult B6 females and males were mated overnight. The first day of gestation was determined by the presence of vaginal plug formation (embryonic day 0.5). On gestation day 12.5 (GD 12.5), pregnant females received a single intraperitoneal dose of 500 mg/kg VPA (Sigma-Aldrich, St. Louis, MO, USA) or 0.9% saline (vehicle) and then were left undisturbed with their male partner and offspring until the time of pup weaning on postnatal day (P) 21. In our study, only male offspring were used (n = 10–12), and behavior tests were conducted at the age of 3 months.

### 4.2. Drug and Treatment

((S)-3-(4-Cyanophenyl)-N-[[1-(3-chloro-4-fluorophenyl)cyclopropyl]methyl]-2-[3-(4-fluorophenyl)ureido]propanamide)—compound MR-39 (CAS Registry Number 2169267-60-9) was synthesized in our laboratories, as previously described [57], dissolved in 20% PEG400, 10% Tween 80, and 70% sterile saline (Sigma-Aldrich, Milan, Italy), and injected at a volume of 10 mg/kg body weight.

BTBR, VPA, and their respective B6 mice, at P90, were subjected to daily intraperitoneal injection (i.p.) up to 8 consecutive days, with vehicle or with the FPR-2 agonist MR-39 at 10 mg/kg (Figure 1A). The dose of FPR2 agonist and the duration of the treatment were selected based on behavioral results shown in the Supplementary material.

### 4.3. Behavioral Tests

On day 8 from the first injection, mice of different cohorts were subjected to behavioral tests. Mice were injected with either MR-39 or vehicle and acclimatized in the experimental room for one hour before performing the test. All behavioral tests were performed during the light period (between 10:00 a.m. and 2:00 p.m.). Data were obtained from three independent cohorts.

#### 4.3.1. Social Approach Test

Social approach behavior was tested in a three-chambered apparatus, using the method previously described [23,85]. The first 5 min of the test is considered the habituation phase, while in the second phase, a novel mouse, matching in sex, strain, and age, is placed in one chamber under a wire cup. The central chamber is the start location, while the third chamber is equipped with an empty wire cup. During the sociability phase, the tendency to approach a novel mouse was compared with the tendency to approach a novel object, monitoring the time spent by the animal in each chamber. Left and right chambers were alternated as mouse-chamber or object-chamber among the subjects. The sociability phase was recorded for 10 min by a video camera coupled with video-tracking software (Any-maze, Stoelting).

#### 4.3.2. Reciprocal Social Interaction Test

Mice interaction was investigated using the reciprocal social interaction test. After a 5-min habituation period in the test chamber, the interactions between the tested mouse and a novel mouse, matching in sex, strain, and age, were recorded for 20 min. The following pro-social interactions were evaluated: push-crawl behavior, nose-to-nose sniffing, anogenital inspection, and self-grooming. The number of events was manually scored. Self-grooming behavior includes head washing, body grooming, genital/tail grooming, and paw and leg licking [86]. After the test, the cage was cleaned thoroughly.

### 4.4. Sample Collection

The day after behavioral tests, mice received the last injection, and after 1 h they were anesthetized and sacrificed by decapitation. The hippocampus was rapidly dissected and immediately frozen at −80 °C for subsequent RNA and protein extraction.

### 4.5. RNA Extraction and Quantification of Gene Expression Using RT-PCR

Total RNA was extracted from hippocampal tissues using TRIzol Reagent (Bio-Rad Laboratories S.r.l, Segrate, Italy) according to the manufacturer’s instructions. cDNA was synthesized using a reverse transcription kit (NucleoSpin^®^, MACHEREY-NAGEL GmbH & Co, Düren, Germany) from 4 µg total RNA. PCRs were performed with a Bio-Rad CFX96 Connect Real-time PCR System instrument and software (Bio-Rad Laboratories). Mouse primers for Tnf-α (Tnf-α), interleukin-1β (Il-1β), interleukin-10 (Il-10), and Glyceraldehyde 3-phosphate dehydrogenase (GAPDH) as housekeeping genes were purchased from Qiagen, Hilden, Germany. Data were analyzed according to the 2^−ΔΔCT^ method.

### 4.6. Enzyme-Linked Immunosorbent Assay

Enzyme-linked immunosorbent assay for LXA4 was carried out on homogenized hippocampus from mouse brain by the LXA4 ELISA kit (MyBioSource Inc., Bergamo, Italy) following the manufacturer’s instructions. Briefly, 100 µL of tissue supernatants, diluted standards, quality controls, and dilution buffer (blank) were applied on a pre-coated plate for 2 h. After washing and incubation with a substrate for the HRP enzyme, the absorbance was measured spectrophotometrically at 450 nm in an iMark microplate reader (Bio-Rad Laboratories S.r.l).

### 4.7. Culturing of Hippocampal Neurons

Primary hippocampal neurons were prepared from BTBR and B6 pups at postnatal day 1 (P1), as previously described [87]. Briefly, brains were isolated under sterile conditions in HBSS. The hippocampi dissected under a stereomicroscope were first placed in HBSS with 10% fetal bovine serum (FBS, Euroclone, Milan, Italy) and then in HBSS w/o serum. The tissues were enzymatically dissociated in a trypsin solution (0.1% trypsin in HBSS) containing 0.01% pancreatic DNAse (Sigma, Milan, Italy) for 1 min and 30 s at 37 °C, and then the reaction was blocked with 10% FBS. Mechanical trituration was performed in the culture medium (see below) containing 0.01% pancreatic DNAse using a fire polished Pasteur pipette. The cells were centrifuged for 3 min at 500 rpm, resuspended in Neurobasal-A medium (Thermo Fisher Scientific, Milan, Italy) containing B27 (Thermo Fisher Scientific), 5% FBS, 2 mM Glutamax (Thermo Fisher Scientific), 50 U/mL penicillin and 50 mg/mL streptomycin (Thermo Fisher Scientific). Hippocampal neurons were plated at a density of 35 × 103/cm^2^ onto sterilized 12 mm coverslips coated with 15 µg/mL poly-D-lysine (Sigma). FBS was withdrawn after 1 day in vitro (DIV), and 5 µM of cytosine b-D-arabinofuranoside (AraC, Sigma) was added at DIV 2 to the culture medium at DIV 2 to inhibit non-neuronal cell proliferation. On the third day in vitro and every 3 days, half of the medium was replaced by fresh medium lacking FBS. For morphological analyses, primary hippocampal neurons at DIV 4 were treated for 4 h and 72 h with 10 µM MR-39. For each experimental point, cultures were prepared from three independent cell preparations.

### 4.8. Immunofluorescence and Morphological Analyses

Neurons in culture were fixed in 4% paraformaldehyde in PBS for 30 min at room temperature. The fixed cells were washed 3 times in PBS and permeabilized for 15 min with 0.1% Triton-X-100 in PBS. After the permeabilization, neurons were treated with blocking solution [3% bovine serum albumin (BSA) in PBS] for 30′ at RT and then incubated with the monoclonal primary antibody against neuron-specific class III ß-tubulin (Tuj1; Sigma-Aldrich T8660, 1:750) overnight at 4 °C followed by 2 h incubation at 22 °C with the fluorescent-labeled secondary antibodies (Alexa Fluor 594, 1:400; Thermo Fisher Scientific) diluted in 3% BSA in PBS. After washing, neurons were stained with 4′,6-diamidino-2-phenulindole (DAPI, nuclear stain, 1:1000) for 10 min at 22 °C and mounted on the coverslip with oil mounting solution (Mowiol, Sigma-Aldrich). Cells processed without primary antibody were used as negative controls.

Images from neurons were acquired using 20× objective and analyzed with a Leica Microscope (Leica DM600B) using the software Leica Application suite. The neurite length was analyzed by the image-processing software Image J and measured as described before [61]. For each cell-culture condition, about 30–35 Tuj1-positive neurons were randomly selected from three independent culture wells. For each neuron, about 2–4 primary neurites that originate directly from soma were traced and measured. Thus, a total of 150 neurites were analyzed for each cell culture condition. To avoid any subjective influences during measurements, the operator blindly analyzed the images.

### 4.9. Statistical Analyses

All data are expressed as the mean ± standard error of the mean (SEM). The statistical significance between the groups was assessed using one-way ANOVA, followed by Tukey’s post hoc test. The criterion for statistical significance was *p* ≤ 0.05. All analyses were performed using GraphPad Prism^®^ 8.4.9 software (GraphPad Software Inc., San Diego, CA, USA).

## 5. Conclusions

In conclusion, using two different mouse models of ASD, we demonstrated that MR-39, a novel FPR2 agonist, rescues LXA4 levels, ameliorates neuroinflammation and social behavior, and stimulates neuronal plasticity, suggesting a causal relationship among these responses. Although the detailed molecular mechanisms underlying the beneficial effects of MR-29 on ASD animal models remain to be investigated, this work is the first demonstration of the involvement of LXA4/FPR2 signaling in ASD mice. Thus, targeting FPR2 might open new scenarios for the treatment of ASD.

## Data Availability

Data is contained within the article and supplementary files.

## Supplementary Materials

The following supporting information can be downloaded at: https://www.mdpi.com/article/10.3390/ph15020161/s1, Figure S1: Dose and time effect of MR-39 on repetitive behavior of BTBR and VPA mice; Figure S2: Dose effect of MR-39 on social behavior of BTBR and VPA mice.
