# Supplementary material for: Behavioral, Anti-Inflammatory, and Neuroprotective Effects of a Novel FPR2 Agonist in Two Mouse Models of Autism
Source: Pharmaceuticals (Basel). 2022 Jan 28;15(2):161. doi: 10.3390/ph15020161 (PMC8875614; doi:10.3390/ph15020161)
Supplement: Supplementary file 1 [file pharmaceuticals-15-00161-s001.zip › pharmaceuticals-1559461-supplementary.pdf]

Supplementary material of *"Behavioral, anti-inflammatory and neuroprotective effects of a novel FPR2 agonist in two mouse models of autism"*

1. Methods

1.1 Marble burying test

Repetitive marble burying was performed allocating each mouse into the test plexiglass container with 20 glass marbles of 1.5 cm in diameter placed on top of 3 cm of clean woodchip bedding, arranged in five rows of four. The animal was allowed to freely explore the cage for 15 minutes. After the test, the mouse was placed back into its home cage, marbles were cleaned, and bedding was replaced with a new one. Each marble was considered buried when it was covered for 75% of its surface.

1.2 Spontaneous self-grooming behavior

Mice were individually allocated in an empty plexiglass cage and allowed to freely explore the arena for 20 minutes. The first 10 minutes served as a habituation period, while during the following 10 minutes the total period of time spent in self-grooming was manually scored by a trained observer. Self-grooming behavior includes head washing, body grooming, genital/tail grooming and paw and leg licking. After the test the cage was cleaned thoroughly.

2. Results

2.1 Effect of FPR2 agonist MR-39 on behavior of BTBR and VPA animals: dose- and time-response.

In order to find the appropriate dose- and time-response effect of MR-39, different doses of the compound (1, 5, 10 and 50 mg/kg) were injected on both mice strains in acute (2 hrs) or chronic regimen (4, 8, 15 days). The animal's behavior was evaluated by marble burying test, self-grooming, and three-chambered social tests. No MR-39 effect was observed in animal models exposed to marble burying test (Figure S1a, $F(19,180)=0.8469$, $p=0.6487$ and Figure S1c, $F(19,180)=0.5627$, $p=0.9282$) and from self-grooming tests (Figure S1b, $F(19,180)=0,9343$, $p=0.5415$, and Figure S1d, $F(19,180)=0.4712$, $p=0.9710$). Thus, we decided to use a chronic treatment of intermediate length, injecting MR-39 i.p. for 8 consecutive days. The dose-response effect of the ligand was evaluated exposing the animal models to the three-chambered social test (Figure S2a, $F(9,70)=8.229$, $p<0.0001$ and Figure S2b, $F(9,70)=7.546$, $p<0.0001$). In this test, we compared the time spent by the animals in the chamber with the novel mouse, to the time spent in the chamber with the empty wire cup (novel object). The vehicle-treated ASD animal models spent more time in the novel object chamber, instead of in the mouse novel chamber, showing a reduced sociability. The treatment with MR-39 induced a significant dose-dependent improvement of sociability: in particular, at the dose of 5mg/kg, mice spent equal time sniffing the novel mouse and the novel object, while, at the doses of 10 mg/kg, they spent significantly more time with the novel mouse than with the novel object. No further significant improvement was observed in mice treated with the dose of 50 mg/kg (Figure S2). Based on these responses, we used for our experiments the dose of 10 mg/kg i.p. injected for 8 consecutive days, which yields the best effects using the lowest dosage.

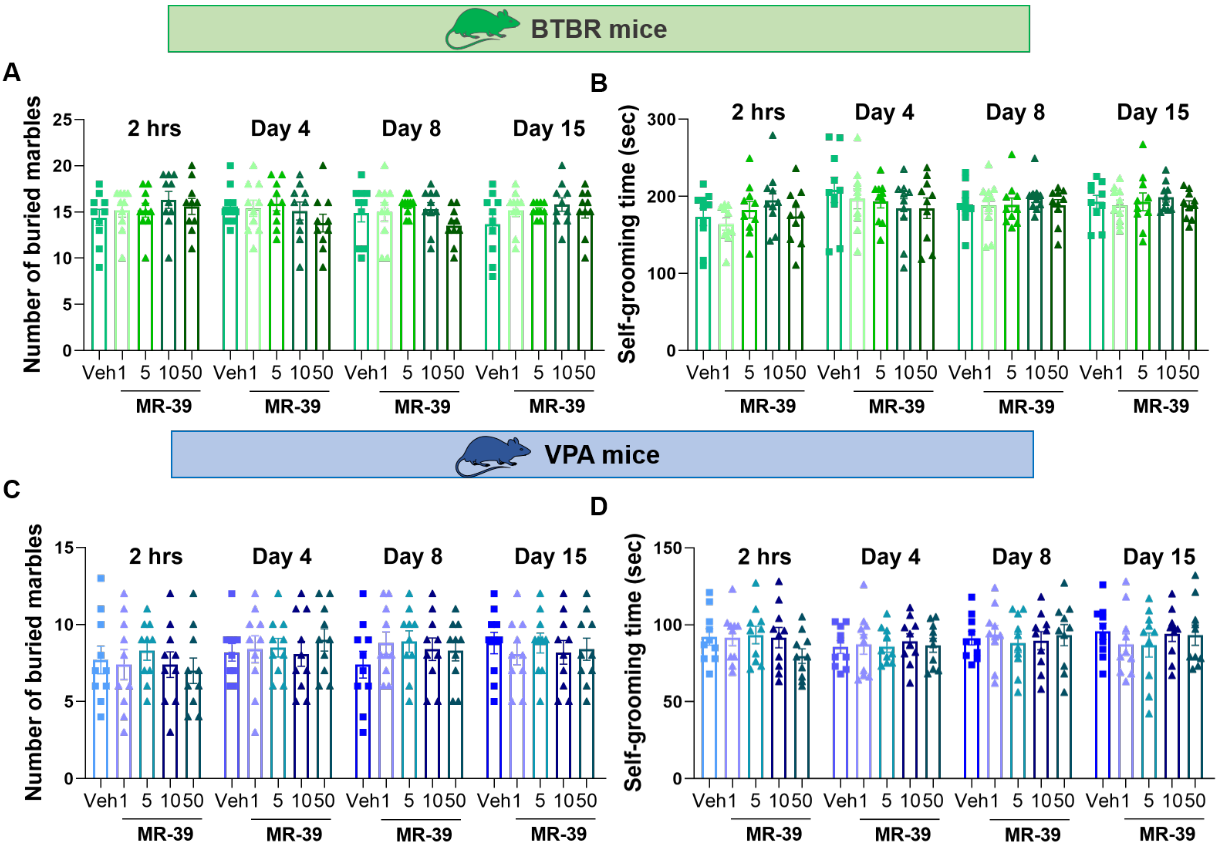

Figure S1: Dose and time effect of MR-39 on repetitive behavior of BTBR and VPA mice. Number of marbles buried, during the 15 minutes test, by BTBR (**a**) or VPA mice (**c**) intraperitoneally injected with different doses (1, 5, 10, 50 mg/kg) of MR-39 or vehicle for 2 hrs, 4, 8 or 15 days. Time (in seconds) spent for self-grooming, during the 10 minutes task, by BTBR (**b**) and VPA mice (**d**) intraperitoneally injected with different doses (1, 5, 10, 50 mg/kg) of MR-39 or vehicle for 2 hrs, 4, 8 or 15 days. Results are shown as means ± s.e.m (n =10).

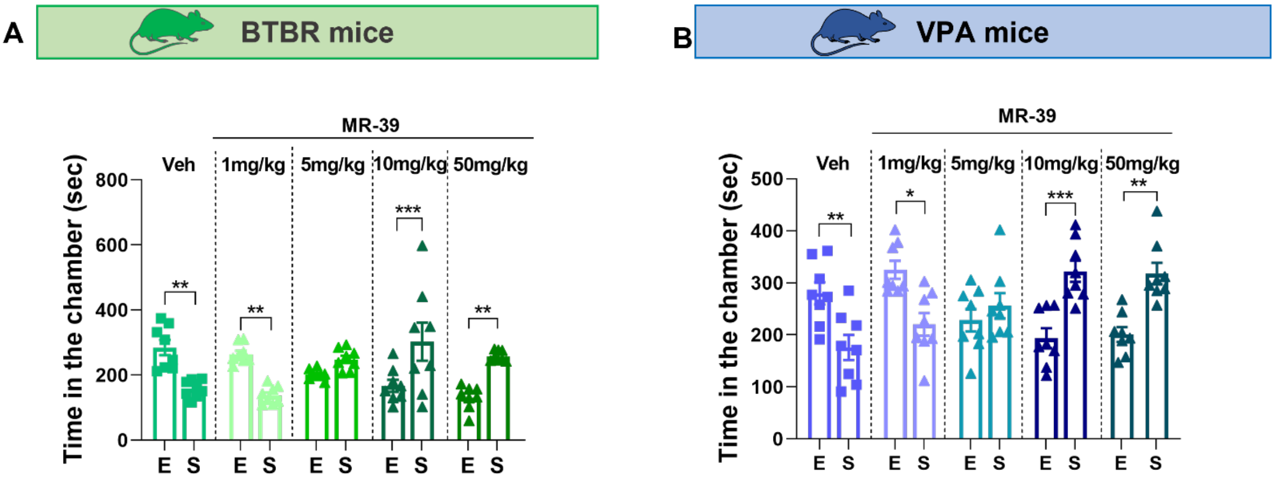

Figure S2: Dose effect of MR-39 on social behavior of BTBR and VPA mice. Time (in seconds) spent in each chamber, during the 10 minutes three-chambered social test, by BTBR (**a**) or VPA mice (**b**) intraperitoneally injected with different doses (1, 5, 10, 50 mg/kg) of MR-39 or vehicle for 8 days. E: chamber with novel object; S: chamber with novel mouse. Results are showed as mean± s.e.m. (n =8). Differences have been evaluated by Student's *t* test, **p*<0.5, ***p*<0.01, ****p*<0.001 vs empty side.
